# Carcinoma developing in ectopic pancreatic tissue in the stomach: a case report

**DOI:** 10.1186/1757-1626-1-249

**Published:** 2008-10-18

**Authors:** Basilios Papaziogas, Ioannis Koutelidakis, Panagiotis Tsiaousis, Konstantina Panagiotopoulou, George Paraskevas, Helena Argiriadou, Stefanos Atmatzidis, Konstantinos Atmatzidis

**Affiliations:** 1 2nd Surgical Clinic, Aristotle's University of Thessaloniki Medical School, "G. Gennimatas" Hospital, Ethnikis Aminis 41, 546 35 Thessaloniki, Greece; 2 Institute of Pathology, "G. Gennimatas" Hospital, Ethnikis Aminis 41, 546 35 Thessaloniki, Greece

## Abstract

The development of pancreatic tissue outside the confines of the main gland, without anatomic or vascular connections between them, is a congenital abnormality referred to as heterotopic pancreas. A heterotopic pancreas in the gastrointestinal tract is usually discovered incidentally and the risk of its malignant transformation is extremely low. In this study, we describe the first case of endoepithelial carcinoma arising in a gastric heterotopic pancreas of a 56-year old woman in Greece. She presented with epigastric pain, periodic nausea and vomiting. Esophagogastroduodenoscopy revealed an ulcerated lesion in the gastric antrum, biopsies of which showed intense epithelial dysplasia with incipient malignant degeneration. The pathology report of the distal gastrectomy specimen demonstrated a 2 cm in diameter ulcerative mass in the gastric antrum. Microscopically, an endoepithelial (in situ) carcinoma of the gastric antrum was determined, which in places turned into an microinvasive endomucosal adenocarcinoma. It also incidentally demonstrated heterotopic pancreatic ducts, detected within the mucosa to the muscularis propria of the same region of the stomach, in which an endoepithelial (in situ) carcinoma was evolving. The follow-up course was uneventful 6 months postoperatively.

## Background

The development of pancreatic tissue outside the confines of the main gland, without anatomic or vascular connections between them, is a congenital abnormality referred to as heterotopic pancreas. It is discovered in approximately 0.5% operations involving the upper abdomen and it is identified in 0.55 – 13.7% of autopsy series [1,2]. Heterotopic pancreatic tissue may be found anywhere from the distal end of the esophagus to the colon in the gastrointestinal tract, but most commonly is found in the stomach (25–40%) [2], mostly in the antrum and prepyloric region on the greater curvature or posterior wall. Unusual sites include the common bile duct, the gallbladder [3], the mesocolon [4], the Meckel's diverticulum [5], the umbilicus [6] and the mesenteric tissue. The heterotopic pancreas is asymptomatic in general, but when discovered incidentally its clinical significance is dependent on the resultant complications. When pancreatic tissue is present in the stomach, symptoms usually resemble those of a mass lesion, with pyloric obstruction, ulceration or bleeding, and they may be accompanied by most of the pathologic changes seen in the eutopic pancreas, e.g. acute and chronic pancreatitis, pseudocyst, abscess. Carcinoma arising in a gastric heterotopic pancreas, though, is extremely rare, with less than twenty cases reported in the literature [2,7-15].

This paper reports the first, to our knowledge, case of endoepithelial carcinoma arising in a gastric heterotopic pancreas of a 56-year old woman in Greece.

## Case presentration

**Figure 1:**
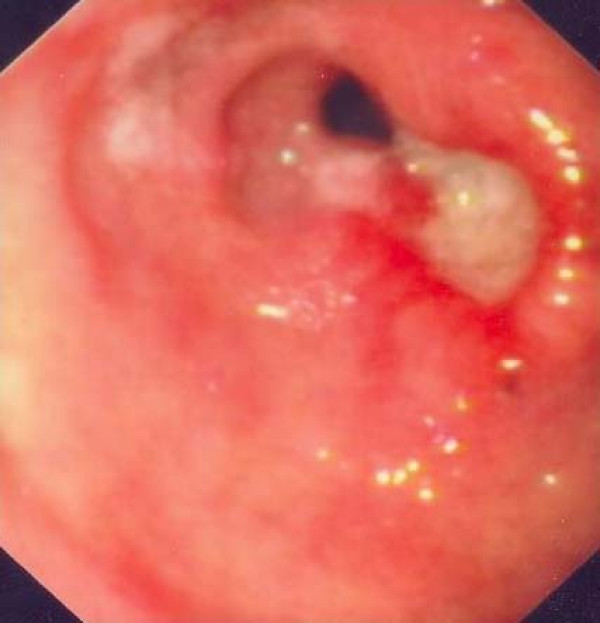
Esophagogastroduodenoscopy: An ulcerated lesion in the prepyloric region.

A 56-year-old woman was admitted because of epigastric pain of several weeks duration, accompanied by periodic nausea and vomiting. Her medical history included a reflux esophagitis with a hiatal hernia and hypothyroidism under medical treatment. On esophagogastroduodenoscopy, an ulcerated lesion in the gastric antrum was found, biopsy specimens of which showed intense epithelial dysplasia with incipient malignant degeneration (Fig. 1). The physical examination and all the laboratory findings were normal. The tumor markers including CEA (1.3 ng/mL), aFP (4.4 ng/mL), CA-19-9 (2.9 u/mL), CA-125 (4.74 u/mL) and CA-72-4 (3.1 u/mL) were within normal ranges. Abdominal ultrasound and thoracic and abdominal CT scan showed no pathologic signs. The patient was electively lead to the operating theatre, where, under a midline incision laparotomy and a subsequent palpation of the stomach, a small prepyloric mass was found. Regional lymph nodes were sent for frozen sections, which showed no signs of malignancy. A distal gastrectomy was performed under the impression of an incipient adenocarcinoma of the gastric antrum.

Gross features of the surgically resected specimen of the distal gastrectomy were of an ulcerated lesion in the gastric antrum, adjacent to the lesser curvature of the stomach, which measured 2 × 1.2 cm, with smooth edges. The rest of the gastric mucosa did not reveal any other macroscopically noticeable findings and the resected margins were free of tumor. Lymph node metastasis was not identified.

**Figure 2:**
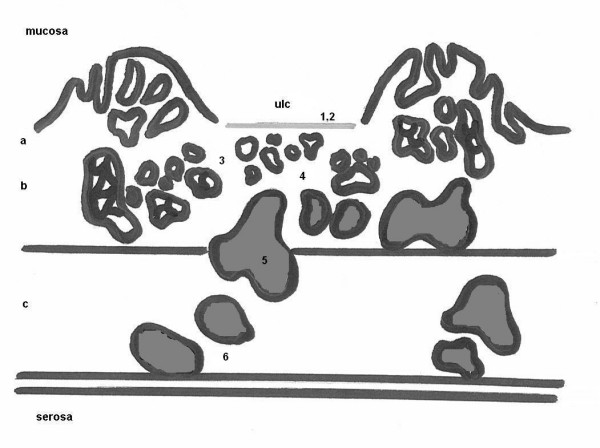
**Schematic diagram of the cut surface of the resected specimen revealing the coexistence of an in situ adenocarcinoma of the gastric antrum with an ectopic pancreas (ducts only) endoepithelial carcinoma. **Numbers correspond to pathology photographs of the surgical specimen seen in Figure 3. Abbreviations: ulc = ulcer, a = mucosa, b = submucosa, c = muscularis propria.

**Figure 3:**
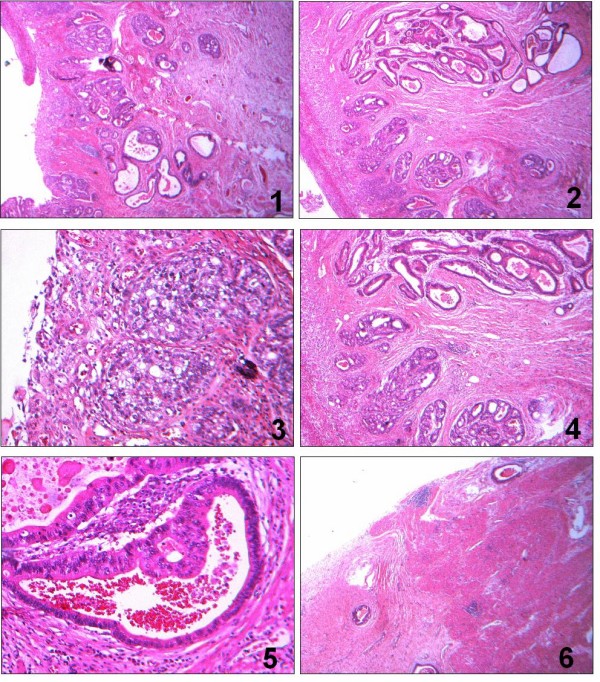
**Pathology photographs of the surgical specimen, which correspond to the numbers noted in Figure 2a.** 1. Ulcerative endomucosal gastric adenocarcinoma (H-E × 6). 2. Ulcerative endomucosal gastric adenocarcinoma and heterotopic pancreatic ducts (H-E × 25). 3. Gastric adenocarcinoma (H-E × 100). 4. Association between gastric adenocarcinoma and heterotopic pancreatic ducts (H-E × 25). 5. Heterotopic pancreatic duct with a dysplastic epithelium (H-E × 100). 6. Heterotopic pancreatic ducts located within the muscularis propria (H-E × 6).

Microscopic findings of the ulcerative gastric lesion of the antrum were characteristic of endoepithelial (in situ) carcinoma (with infiltrating morphology in most of its area) (Fig. 2a and 3). Small gastric microinvasive mucosal adenocarcinoma foci were recognized in few surface sites. In the same gastric region ectopic pancreatic ducts were located within the mucosa, submucosa and muscle layer (3/4 of its depth). Their epithelium was either normal or dysplastic at places, a dysplasia which varied from mild to significant, assuming in many cases characters of endoepithelial (in situ) carcinoma. No disruption of the lamina propria of the ectopic ducts was noticed. The gastric mucosa adjacent to the lesion presented with extensive enteric metaplasia, dysplastic vitiations and a few ectopic ducts. The resected margins and the omentum were free of tumor. Lymph node metastatic infiltration was not identified.

The pathological findings lead us to the conclusion that we were dealing with a gastric endoepithelial (in situ) carcinoma, which at places assumed characters of a microinvasive endomucosal adenocarcinoma. It also incidentally demonstrated heterotopic pancreatic ducts, detected within the mucosa to the muscularis propria of the same region of the stomach, in which an endoepithelial (in situ) car inoma was evolving.

We then carried out immunohistochemical staining using the antibodies shown in Table 1. In the area of gastric tumor tissue cytokeratin (CK) 7 and Ki-67 had the strongest positive immunoreactivity, while cytokeratin 7 and carcinoembryonic antigen (CEA) were almost equally positive in the heterotopic pancreatic ducts site.

**Table 1:** Immunohistochemical staining pattern in the present case

Antibody	Gastric tumor site	Gastric heterotopic pancreas site	Description & use of antibody
CEA	(+)	(++)	Oncofetal antigen in gastrointestinal, breast, lung, pancreas and other cancers.
			
CA 19-9	(-)	(-)	Useful cerum marker in gastrointestinal and pancreatic cancer; strongest staining in pancreatic cancer.
			
Cytokeratin 7	(+)	(++)	Typically found in simple epithelia from the GI tract; lesser degree of expression in gastric cancer.
			
Cytokeratin 19	(+)	(-)	Cytokeratin filament in a large number of epithelial cell types, including many ductal and glandular epithelia.
			
Cytokeratin 20	(±)	(-)	Restricted expression in intestinal epithelium, gastric foveolar epithelium and others; lesser degree of expression in pancreatic cancer.
			
MUC1	(-)	(+)	Glycoprotein of glandular epithelial tissues; modifications to glycosylation of musins in cancer; "gastric mucin" & "PanINs mucin".
			
Ki-67	(++)	(-)	The assessment of cell proliferation in neoplastic cell populations

CA, carbohydrate antigen; CEA, carcinoembryonic antigen; MUC, mucin (transmembrane glycoprotein tumor antigen); -, < 5% cells stained; +, 30–70% cells stained; ++, > 70% cells stained; ±, 5–30% cells stained; GI, gastrointestinal; PanIns, pancreatic intraepithelial neoplasias

Immediate and late (after six months) postoperative course of the patient was uneventful.

## Discussion

Heterotopic (ectopic, aberrant) pancreas is frequently found in the gastrointestinal tract, especially in the antrum of the stomach. Shultz was first to describe heterotopic pancreas in 1727, although histological data were not available until the report by Klob in 1859, in which the latter presented two cases of heterotopic pancreas, one of which was located in the stomach [1]. However, malignant change in heterotopic pancreas is extremely rare, and there are not many published reports of cases of gastric carcinoma presumed to arise from heterotopic pancreatic tissue in the gastric wall [2,7-15]. To our knowledge, Pforringer [16] first reported in 1904 an association of gastric carcinoma with heterotopic pancreas.

The heterotopic pancreas may contain any mixture of tissues normally found in the pancreas. Heinrich classified the heterotopic pancreas into 3 types [17]: type I, all the components of the pancreas including ducts, acini, and endocrine islets; type II, ducts with acini; and type III, ducts with a few acini or dilated ducts only, so called adenomyoma. When the pancreaticobiliary-type ducts predominate, they are often surrounded by hypertrophic smooth muscle bundles. The present case belongs to the Heinrich type III (adenomyoma) ectopic pancreas, showing dilated pancreaticobiliary-type ducts only.

The typical appearance of lesions of gastric heterotopic pancreas as supported by endoscopic observation is that of polypoid submucosal or muscularis growths occasionally displaying a central umbilication, which may be the site of ductal drainage to the mucosal surface. The umbilication is observed in less than half of cases, therefore, other intramural masses such as gastrointestinal stromal tumor or even metastatic carcinoma must be differentiated. The tissue appears as discrete yellowish-gray nodules with well-defined lobules of acinar tissue that may be replete with islands of Langerhans as well as exocrine glands. These nodules are usually small, measuring 3 cm or less in diameter, although the lesions in the stomach are usually larger than in other sites, averaging 2.4 cm.

For a carcinoma to be described as arising from heterotopic pancreatic tissue, three criteria have been proposed [16,12]. Firstly, the tumor must be found within, or close to the aberrant pancreatic tissue. Secondly, the transitional area between pancreatic structures and carcinoma must be observed (i.e. duct-cell dysplasia and/or carcinoma in situ). Thirdly, the non-neoplastic heterotopic pancreatic tissue must comprise of at least fully developed acini and/or ductal structures. The lesion in the present case had findings compatible with all three criteria.

With regards to immunohistochemical findings, the staining pattern of this specimen wasn't explicit enough to differentiate whether the tumor resembled that of a primary adenocarcinoma arising from heterotopic pancreas or that of an adenocarcinoma arising from gastric epithelium and secondarily infiltrating the gastric heterotopic pancreatic tissue. The similar CK7+/CK20- phenotype in both the gastric in situ adenocarcinoma site and the gastric heterotopic pancreas in situ carcinoma site doesn't correspond to the study by Duval et al. [13], which showed the CK7+/CK2O- phenotype in about 96% carcinomas of the pancreas.

Most cases of gastric heterotopic pancreas are discovered (1) as an incidental finding on upper gastrointestinal series, (2) at operation, coincidental to some other pathological condition contributing to the surgical indications or (3) at autopsy. Thus, this entity is usually unrecognized clinically and its clinical course is generally uneventful. Asymptomatic lesions of less than 2 cm can be followed without specific therapy. However, the combination of a clinical presentation consisting of weight loss, nausea and vomiting, and an ulcerating lesion in the upper gastrointestinal endoscopic examination, as in our case, should raise a suspicion of malignant transformation. Therefore, all lesions of gastric heterotopic pancreas that are symptomatic (particularly with bleeding or obstruction, or are found to be larger than 3 cm in size, regardless of the symptoms, should be treated with surgical resection with histologic confirmation. For asymptomatic small lesions less than 2 cm in size, only local resection can be considered, although histologic confirmation is required at the operation field by frozen section, to decide the extent of surgery. The clinical course of cases of carcinoma within gastric heterotopic pancreas, despite the small number of reported cases, appears to parallel that of primary gastric cancer and, therefore, the management is considered to be the same [2].

The coexistence of an adenocarcinoma of the stomach and an ectopic pancreas (ducts only) with dysplasia and endoepithelial carcinoma gives birth to a crucial question. Are these two malignancies independent and co-existing or the infiltration of an adenocarcinoma of the gastric epithelium to the gastric ectopic pancreatic ducts? Although the immunohistochemical findings with CK-7, CK-20, CK-19, CA 19-9, CEA, MUC-1 and Ki-67 weren't explicit enough to differentiate the case, the presence of a transitional phase (from normal to dysplastic and to an in situ carcinoma) in the epithelium of the heterotopic pancreatic ducts leads to the conclusion that the carcinoma is a pancreatic one.

## Competing interests

The authors declare that they have no competing interests.

## Authors' contributions

BP was in the operating team and had the responsibility for the writing of the manuscript, IK was in the operating team and significantly contributed to the writing of the manuscript, PT significantly contributed to the literature research histological examination of the specimen and writing of the manuscript, KP perfomed the histological evaluation of the specimen, GP was in the operating team, HA significantly contributed to the writing and linguistic formatting of the manuscript, SA significantly contributed to the clinical management of the patient postoperatively and the literature research, KA was in the operating team and significantly contributed to the correction of the manuscript. All authors read and approved the final manuscript."

## Consent

We have obtained written, informed consent from the patient in order to publish this case report.
